# Exploiting Nanomedicine for Cancer Polychemotherapy: Recent Advances and Clinical Applications

**DOI:** 10.3390/pharmaceutics15030937

**Published:** 2023-03-14

**Authors:** Elena Boggio, Casimiro Luca Gigliotti, Ian Stoppa, Deepika Pantham, Sara Sacchetti, Roberta Rolla, Margherita Grattarola, Chiara Monge, Stefania Pizzimenti, Umberto Dianzani, Chiara Dianzani, Luigi Battaglia

**Affiliations:** 1 Dipartimento di Scienze della Salute, Università del Piemonte Orientale, 28100 Novara, Italy; 2 Ospedale Universitario Maggiore della Carità, 28100 Novara, Italy; 3 Dipartimento di Scienze Cliniche e Biologiche, Università degli Studi di Torino, Corso Raffaello 30, 10125 Torino, Italy; 4 Dipartimento di Scienza e Tecnologia del Farmaco, Università degli Studi di Torino, 10125 Torino, Italy; 5 Centro Interdipartimentale Nanostructured Interfaces and Surfaces (NIS) Interdepartmental Centre, Università degli Studi di Torino, 10124 Torino, Italy

**Keywords:** polychemotherapy, nanocarriers, cancer therapy

## Abstract

The most important limitations of chemotherapeutic agents are severe side effects and the development of multi-drug resistance. Recently, the clinical successes achieved with immunotherapy have revolutionized the treatment of several advanced-stage malignancies, but most patients do not respond and many of them develop immune-related adverse events. Loading synergistic combinations of different anti-tumor drugs in nanocarriers may enhance their efficacy and reduce life-threatening toxicities. Thereafter, nanomedicines may synergize with pharmacological, immunological, and physical combined treatments, and should be increasingly integrated in multimodal combination therapy regimens. The goal of this manuscript is to provide better understanding and key considerations for developing new combined nanomedicines and nanotheranostics. We will clarify the potential of combined nanomedicine strategies that are designed to target different steps of the cancer growth as well as its microenvironment and immunity interactions. Moreover, we will describe relevant experiments in animal models and discuss issues raised by translation in the human setting.

## 1. Introduction

Cancer cells counteract the stress induced by anticancer drugs with several mechanisms, including (1) mutations of oncogenes and tumor suppressor genes, (2) changes in cellular drug import and export, (3) increase in DNA damage repair mechanisms, (4) evasion from apoptosis, and (5) adaptation to oxidative stress [1]. Patients with the same tumor can display different responses to the same therapy. This heterogeneity is due to tumor genome instability caused by mutations and to complex chromosome rearrangements [2]. Tumor heterogeneity also brings resistance to targeted therapy [3], involving not only the genetic and nongenetic modifications of the target molecules and their signaling pathways, but also the modulation of different compensatory pathways [4].

**Figure 1:**
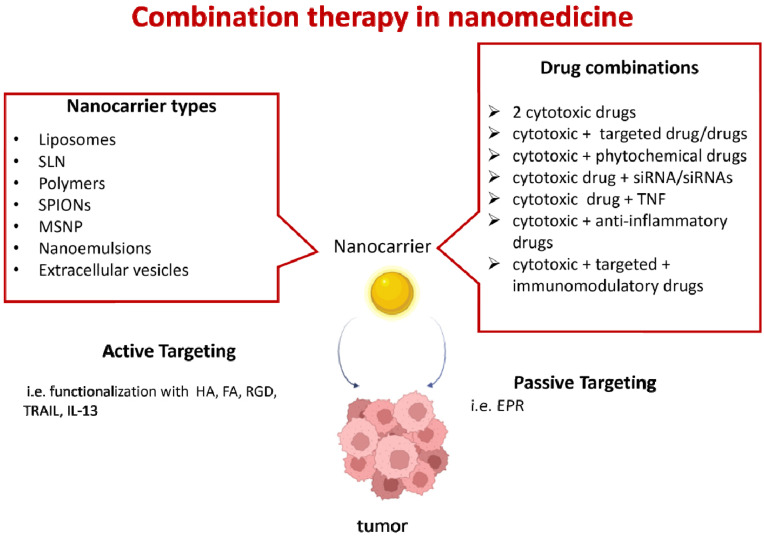
Combination therapy in nanomedicine. Several types of nanocarriers can be used: liposomes; solid lipid nanoparticles (SLN); polymeric nanoparticles (i.e., poly(ethylene glycol)-block-poly(2-methyl-2-benzoxycarbonylpropylene carbonate); polyethyleneimine-block-poly[(1,4-butanediol)-diacrylate-β-5-hydroxyamylamine]; polyethylene glycol-block-poly[(1,4-butanediol)-diacrylate-β-5-hydroxyamylamine]; or poly lactic-co-glycolic acid (PLGA); superparamagnetic iron oxide (SPIONs); mesoporous silica nanoparticles (MSNP); nanoemulsions; or extracellular vesicles. Drug combinations can include cytotoxic drugs (i.e., doxorubicin, vincristine, camptothecin, paclitaxel, and temozolomide); targeted drugs (i.e., the tyrosine kinase inhibitors erlotinib, lapatinib, and sorafenib); trastuzumab antibodies targeting the human epidermal growth factor receptor 2 (HER2); the mammalian target of rapamycin (mTOR) inhibitor rapamycin; and bevacizumab, a humanized anti-VEGF monoclonal antibody; siRNAs (i.e., siRNAs targeting the epithelial–mesenchymal transition, such as Snail and Twist); or anti-apoptotic molecules (such as Bcl-2); phytochemicals with anticancer properties (i.e., quercetin); tumor necrosis factor (TNF) superfamily molecules (i.e., tumor-necrosis-factor-related apoptosis-inducing ligand—TRAIL); immunomodulatory drugs (i.e., ICOS-Fc); and anti-inflammatory drugs (i.e., aspirin). Active or passive targeting can be used. This figure was partially created with BioRender.

Combination therapy is clinically effective for cancer treatment; the synergistic effects allow a reduction in the doses and the systemic toxicity of each drug and can suppress multi-drug resistance (MDR), which occurs mainly due to P-glycoprotein (P-gp)-mediated extrusion of xenobiotics from tumor cells [5]. Moreover, combination therapy increases the chances of targeting cancer stem cells, which play a key role in cancer initiation, invasion, metastasis, and recurrence. Finally, combination therapy simultaneously targets the cancer microenvironment; for instance, by inhibiting cancer angiogenesis and promoting the anticancer immune response [6,7]. However, combination therapies need to be optimized, because not all drugs can work together due to the differences in their pharmacokinetic and biodistribution profiles [8]. Furthermore, the association of chemotherapeutic drugs [9] and innovative MDR chemosensitizers is still limited by solubility, stability, and bioavailability issues, as well as by their therapeutic indexes [10,11] (Figure 1).

## 2. Advantages and Challenges of Combined Nanomedicines

### 2.1. Rationale and Design

Nanocarriers show several advantages for the efficient loading of anticancer combination therapies. According to the European Medicines Agency (EMA), the term “nanomedicines” includes all “structures” with a size smaller than 1000 nm designed to have specific properties and to improve site-specific drug delivery and/or toxicological profiles [12]. Combined nanomedicines can load multiple drugs, overcoming their solubility issues and separating incompatible compounds into physically distinct environments. Their multiple cargoes can be driven to the same target tissue, where several cellular mechanisms can be addressed synergistically, reducing the therapeutic dose of each drug. In some cases, these nanomedicines might be internalized at the cellular level together with their cargo, preventing cell extrusion mechanisms involved in MDR and, thus, increasing the therapeutic efficacy [5]. 

Nonetheless, some relevant issues should be considered. 

Firstly, co-encapsulation of drugs in the nanocarrier should occur in a precise, therapeutically synergistic ratio [11]. To this aim, the loading and/or surface absorption of drugs in nanocarriers can be tuned according to their lipophilicity, solubility profile, molecular weight, and chemical interaction (hydrophobic, electrostatic, and/or covalent) with a certain moiety, and made susceptible to further modification without loss of activity. Suitable chemical strategies can be used, including drug hydrophobic ion pairing with oppositely charged surfactants, polymer-drug complexes, and cleavable prodrugs [13]. Within this concern, vesicular systems show several advantages. Indeed, liposomes can be loaded with either hydrophilic or hydrophobic drugs, in the inner aqueous core and in the lipid by-layer, respectively. A similar behavior can be achieved with polymersomes, which are polymeric vesicles composed of self-assembled amphiphilic block copolymers with an aqueous core and a polymer shell. Increasing the membrane thickness of the polymersomes increases also their stability, rigidity, and entrapment of hydrophobic drugs [14]. 

Secondly, loading multiple drugs into a nanocarrier can lead to oversized nanomedicines, which are quickly removed from the bloodstream by the reticulo-endothelial system (RES) and are, therefore, unable to efficiently reach the tumor tissue. In order to manage this concern, versatile formulation processes can be employed, such as layer-by-layer assembly [8]. In this work, an 8 nm iron oxide core was coated with negatively charged silica-polyethylene glycol (PEG) and subsequently conjugated with a peptide targeting tumor vessels (endoglin-binding peptide), a chemotherapeutic drug (doxorubicin), and an immunomodulating agent (Poly IC).

Finally, the drug release from “combined” nanomedicines should be modulated depending on their targets in the tumor microenvironment. For instance, in order to suppress MDR, chemosensitizers should be released before the MDR substrates. Burst release of the chemosensitizer can be achieved using a composite nano-structure [5,8,15,16] or by exploiting the different affinities of the loaded compounds for the nanocarrier matrix [17] and/or the different features of the physiological versus tumor environment, such as the acidic pH of the tumor microenvironment [18].

Nanocarriers may also allow combining gene and chemotherapies synergistically, which may hamper chemoresistance due to MDR [19] or DNA repair [20]. These nanocarriers should have high transfection capabilities (in the absence of toxicity) and a synergistic drug payload [21]. In order to deliver nucleic acids successfully, such nanomedicines should overcome the intracellular barriers, including endo-lysosomal degradation, and, in the case of plasmid DNA, they should also cross the nuclear membrane [22]. In order to avoid unwanted interactions between the chemotherapeutics and nucleic acids, loading within the nanocarrier is done sequentially (usually drugs first, followed by the nucleic acid), even if some studies describe a co-loading strategy using dedicated precautions [14]. Electrostatic interactions are often exploited to condense nucleic acids onto the surface of classical cationic nanocarriers, but recently, covalent linkage between a nucleic acid and the polymer matrix was shown to allow encapsulation of large amounts of nucleobases and to simultaneously load hydrophobic drugs within the polymer core [23]. In this case, the novel synthetic DNA analog click nucleic acids (CNAs), PEG, and poly(lactic-co-glycolic acid) (PLGA) were used to produce nanoparticles (NPs) based on PEG-CNA-PLGA triblock copolymers, capable of encapsulating a large amount of DNA containing an overhang complementary to the CNA sequence together with the hydrophobic drugs. Combination therapy frequently includes short interfering RNA (siRNA). Interestingly, Chen S, et al. [24] loaded two siRNAs, one for vascular endothelial growth factor receptor 2 (VEGFR2) and one for epidermal growth factor receptor (EGFR), in NPs based on polyethylenimine (PEI), a cationic polymer widely studied as a vehicle for nucleic acids. These NPs were used to treat non-small-cell lung cancer (NSCLC), either in the presence or in the absence of cisplatin (CIS). Low-dose CIS, combined with the two siRNAs, induced a reduction in tumor growth and increased survival of A549-injected Balb/c nu/nu mice.

Within this context, an innovative approach is represented by hybrid nanomaterials, which are defined as composites of at least two constituents, usually organic and inorganic, at the nanometer or molecular level, that are engineered to integrate the advantages of biocompatibility and functionality endowed by organic and inorganic components and/or to reveal new properties as a result of this hybridization [25,26]. Moreover, multiple diagnostic and therapeutic nanocomponents can be integrated in a “theranostic” device. Several preparation strategies have been reported, exploiting covalent and noncovalent conjugation, which can be broadly classified as resembling goods-carrying methods on ships: (1) stacking the cargo on the deck of a barge or (2) placing it in the closed container of a tanker. Liposomal, micellar, porous silica, polymeric, viral, noble metal, and nanotube systems can be incorporated either within a porous nanostructure (“tanker”), or grafted at the surface of a nanoparticle (“barge”). The choice between these two strategies depends on the nanocargo and the delivery requirements. Indeed, a reactive or antigenic drug should be protected from the biological environment in a “tanker” until it reaches its target, whereas an imaging agent grafted to the external surface of a “barge” can be more readily accessed and/or rapidly released in response to physiological stimuli. For a large carrier sphere, many more molecules can be contained inside the “tanker” than grafted on the “barge” surface, whereas for a small carrier sphere, more molecules can be placed on its surface than contained inside because the interior space available to load drugs is decreased. The cutoff size for the nanoparticle carrier is tenfold the size of the molecular payload: that is, nearly 20 nm for small compounds and 100 nm for large proteins. Advantageously, the “tanker” approach avoids chemical modification of the loaded drugs, which is required for the surface-loaded “barge” nanocarriers. This represents a critical issue for regulatory approval because a chemically modified drug is considered a new chemical entity [25].

### 2.2. Claimed Advantages

Combined nanomedicines can be synthesized using several types of matrixes (inorganic, metal, polymeric, lipid, and surfactant) and various supramolecular structures (dendrimers, vesicles, nanoemulsions, core-shell nanocapsules, and nanospheres) [27,28] by employing bottom-up and top-down approaches, depending on whether the building blocks are added onto a substrate or removed from it, respectively [29]. Two or more types of materials can be integrated to obtain nanocarriers with optimized physicochemical properties, enhanced targeted delivery, and controlled drug release. Synthetic polymers allow easy fabrication and functionalization as well as the absence of biological contamination; pure metals or metal oxides combine in vivo multimodal imaging and therapy; natural polymers are characterized by biodegradability [12]. However, owing to their biocompatibility, lipid-based nanoformulations, liposomes, nanoemulsions, and solid lipid nanoparticles (SLN) are the nanocarriers most employed. In particular, liposomes and nanoemulsions have a long history of safe clinical usage for drug delivery and total parenteral nutrition [30].

Moreover, several advantages are specifically associated with hybrid nanomaterials in cancer therapy, even though they have just entered biomedical use in recent decades, and therefore, few studies regarding their biosafety in different animal species are available to date. Although chemical synthesis and self-assembly are the main nanoparticle production approaches to date, bioengineering is becoming prominent for the construction and screening of novel biologically-based hybrid nanomaterials, such as those acting on emerging forms of cancer cell death (apoptosis, autophagy, ferroptosis, and pyroptosis) [26]. Moreover, activation of innate and adaptive immunity can be achieved with nanoscale metal–organic frameworks (nMOFs), whose porous structure can be easily loaded with immunologic proteins and immune adjuvants, through a biomineralization self-assembling process [31].

### 2.3. Tumor Targeting 

Specific advantages of combined nanomedicines arise from the nanocarrier targeting capability that can be achieved owing to passive, active [8,10,32], or stimuli-responsive mechanisms [33,34]. Passive targeting depends on the enhanced permeation and retention (EPR) effect, which requires small nanocarriers (<200 nm) and a hydrophilic coating to avoid RES opsonization [13]. This allows extravasation and accumulation of the NPs in the tumor tissue, which is associated with a leaky vascularization. The tumor targeting can be improved when the EPR effect is combined with active targeting strategies such as functionalization with antibodies, antibody fragments, and peptides that are specific for the tumor microenvironment [35,36,37,38]. However, different critical issues are associated with active targeting, including the poor selectivity of available ligands for the target tissue, scarce ligand–receptor interaction (which might be overcome by using spacers between the nanocarrier and ligand), and immunogenicity, in the case of protein ligands [39]. Among the stimuli-responsive approaches, nanomaterials responsive to an acid pH are particularly relevant because a rapid release of the cargo at acid pH allows its selective accumulation in the acidic tumor microenvironment and its endo-lysosomal escape at the cellular level, which is relevant for efficient gene delivery [19,20]. This is also important to hamper MDR, because resistant cells often display an enhanced sequestration of therapeutic agents in endo-lysosomes [20]. 

**Figure 2:**
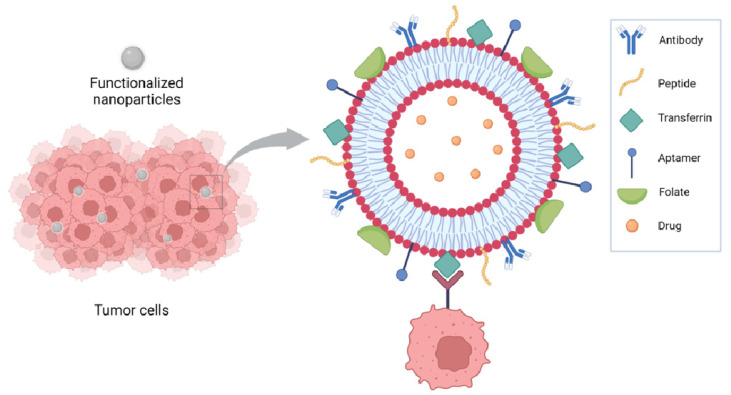
Functionalization of liposomes has been achieved using several approaches including peptides, antibodies, small molecules, and aptamers.

Functionalization of nanoparticles for delivery of anticancer drugs has been extensively reviewed by Seidu et al. [40]. Focusing on liposomes, NP functionalization has been achieved using several approaches including peptides, antibodies, small molecules, and aptamers. Antibodies have been used as either whole antibodies or antibody fragments, such as a single-chain fragment variable (scFv) targeting the receptors expressed on tumor cells such as CD133 for glioblastoma, and the carbonic anhydrase overexpressed in several solid tumors because of the hypoxia in lung cancer. HER2, overexpressed in breast cancer, has been targeted using antibodies and affibodies, which are antibody mimetic proteins. Transferrin receptors, overexpressed in several tumors, have been targeted using either antibody fragments or peptides such as T7 peptide or transferrin itself; RGD peptides have been used to target integrins and folate to target folate receptors. Aptamers are synthetic oligonucleotides capable of binding to specific targets; they have been used to target molecules overexpressed on the tumor cell surface, such as nucleolins and CD44, or in the tumor microenvironment, such as IL-4Rα [40] (Figure 2).

An alternative approach has been the use of stimuli-responsive liposomes releasing their contents into the tumor mass in response to stimuli such as temperature, pH, light, magnetic fields, ultrasound, and redox [41].

### 2.4. Limitations and Hurdles

Several hurdles are associated with nanomedicines, including a low drug payload, meaning that only potent drugs can be employed successfully, and high volumes should be administered in order to reach a therapeutic dose; stressful synthetic conditions (heat, extreme pH, solvents, highly reactive components, and so on) that are potentially harmful to sensitive drugs; toxicological concerns, especially for poorly explored inorganic nanocarriers; and scale-up issues, especially for actively targeted nanomedicines [12]. Moreover, the presence of residual solvents, reaction byproducts, and endotoxins could enhance cellular toxicity and require extensive purification processes [42]. Dose timing is a critical factor in the delivery of drug combinations because simultaneous drug delivery can result in both synergy and antagonism. For instance, liposomes co-loaded with fluoroorotic acid and irinotecan were more efficacious than co-administration of liposomes loaded with the single drugs in the same ratio, whereas significant antagonism was reported between doxorubicin and vincristine co-loaded in the same liposomes [43]. It is noteworthy that the co-encapsulation process follows its own ratios, which may not match with the ones required for synergism. Moreover, some drug pairs are not suitable for co-encapsulation because in the nanocarrier core, spatial proximity may trigger a wide array of physicochemical interactions that may alter the biochemical attributes of the encapsulated drugs in an unpredictable manner, also impacting the release kinetics and/or affecting their colloidal stability. This is one reason why an adequate batch-to-batch reproducibility of combined nanomedicines is still a synthetic challenge [44]. Challenges associated with co-delivery might also drive toward the use of nanodevices for just one of the combined drugs, especially for molecules whose limitations can hardly be sorted out using nanotechnology, as documented for immune checkpoint inhibitors co-administered with nanocarrier-loaded chemotherapy, in order to obtain synergism [45,46,47].

All these factors strongly limit the successful overcoming of the so-called vitro/vivo bottleneck for combined nanomedicines as well as the human translation, which firstly depends on the assessed efficacy on appropriate animal models able to replicate the human condition [48]. Thus, except for Vyxeos, a co-encapsulated liposomal formulation of daunorubicin and cytarabine indicated for myelodysplasia-related acute myeloid leukemia, no other combined nanomedicine has gained approval so far [44]. Therefore, this manuscript depicts the current landscape of combined nanomedicines in three steps: firstly, by summarizing the most relevant studies to date involving conventional nanocarriers that achieved successful preclinical evaluation either in tumor animal models or at least on three-dimensional tumor models capable of adequately simulating the pathological condition; secondly, by elucidating the main issues encountered by such formulations when entering clinical trials; thirdly, by describing innovative nanocarriers, which show promising translation potential compared with other NPs due to their enhanced targeted delivery and drug loading and their controlled release capability.

## 3. Relevant Studies on Animal Models

On December 2022, a Pubmed search on “nanoparticles cancer therapy” obtained more than 25,000 results, which was restricted to about 5000 by adding “breast”; nearly 1000 by adding “melanoma”, “colon”, or “prostate”; about 650 by adding “glioblastoma”; and 100 by adding “pancreas”. Therefore, we decided to focus our review on the main approaches involving combined nanomedicine to target breast cancer, as the top scoring cancer; melanoma, as the prototypical highly immunogenic tumor; and glioblastoma, as the most invasive central nervous tumor. Relevant results on tumor animal models were considered as the threshold for inclusion.

### 3.1. Combined Nanomedicines to Treat Breast Cancer

Breast cancer is the leading cause of death among women; in particular, the triple-negative (TBNC) variant is characterized by a bad prognosis due to its resistance to conventional drugs [49] and strongly requires novel therapeutic approaches. To deliver combined therapy, several types of NPs have been used, including lipid, polymeric, and inorganic NPs. 

Among traditional chemotherapeutics, doxorubicin (DOX) plays a key role: combined nanomedicines were frequently employed to overcome DOX chemoresistance in TBNC breast cancer. Liposome-based nanocarriers loaded with DOX in combination with erlotinib (ERLO), an EGFR (epidermal growth factor receptor) inhibitor, was significantly able to reduce tumor growth in a xenograft mouse model obtained with the BT-20 human breast cancer cell line, by promoting apoptotic signals and tumor cell death [50]. SLN loaded with DOX combined with alpha-tocopherol succinate also displayed increased anti-tumor activity in a breast cancer model [51]. In order to enhance the cytotoxic properties of DOX and overcome MDR, Wang H et al. [52] used poly(ethylene glycol)-block-poly(2-methyl-2-benzoxycarbonylpropylene carbonate)-based NP containing DOX and lapatinib, a kinase inhibitor, in order to treat the drug-resistant breast cancer induced with MCF-7/ADR cells in xenogenic mice. The combined drug administration was more effective in reducing tumor growth than DOX alone. Moreover, hyaluronic acid (HA) NPs loaded with DOX and CIS [53] were effective at lower dosages than those used for the single drugs in reducing the tumor growth induced by the injection of MDA-MB-468LN cells in nude mice. 

However, the most efficient active targeting for combined nanomedicines in breast cancer was achieved by means of trastuzumab monoclonal antibodies (brand name Herceptin), targeting the human epidermal growth factor receptor 2 (HER2), which have been tested in vivo in orthotopic mouse cancers induced with TBNC 4T1 and HER2-positive SKBR3 cells. Results showed that these immunoliposomes effectively reduced tumor growth in the 4T1 model and drug IC_50_ in the SKBR3 model [54]. The NP consisting of a mucic acid polymer loaded with camptothecin (CPT) and a single Herceptin molecule per NP [55] showed complete inhibition of tumor growth compared with the single drugs or CPT-loaded NPs without the antibody. 

Mitotic fuse inhibitors were successfully employed in combined nanomedicines. Polymeric methoxy poly (ethylene glycol)-poly (lactide-co-glycolide) (MPEG-PLA) nanocarriers loaded with paclitaxel (PTX) and gemcitabine (GEM) displayed decreased breast cancer growth and reduced systemic toxicity compared with the free drugs in tumors induced with 4T1 cells in Balb/c mice. These biodegradable polymeric NPs were characterized by a low clearance rate and an ability to optimize the drugs’ pharmacokinetic profiles [18,56]. Liposome-delivered formulations loaded with vincristine and co-encapsulated with quercetin displayed enhanced activity in a trastuzumab-insensitive xenograft model [57]. 

Finally, siRNA was frequently combined with traditional chemotherapeutic drugs in composite nanomedicines to treat breast cancer. A complex system has been developed based on two amphiphilic polymers containing two different siRNAs, one against Snail and the other against Twist, two well-known epithelial–mesenchymal transition (EMT) regulators, in combination with PTX [58]. The polymers used were polyethyleneimine-block-poly[(1,4-butanediol)-diacrylate-β-5-hydroxyamylamine] and polyethylene glycol-block-poly[(1,4-butanediol)-diacrylate-β-5-hydroxyamylamine]. Such NPs reduced the growth of tumors induced with 4T1 cells more effectively than the same NPs containing each siRNA alone. Moreover, this treatment decreased the development of metastases, owing to reduced cell motility, suggesting its possible use for treatment of metastatic breast cancer. Superparamagnetic iron oxide NPs (SPIONs) coated with polymeric trimethyl chitosan (TMC) and functionalized with folic acid (FA) were used for co-delivery of siRNAs against EZH2 and CD73 in order to treat the 4T1 cancer model [59]. TMC was exploited because it favors the binding of oligonucleotides and FA because its receptor was highly expressed by the tumor cells and thus it increased the uptake by the tumor. CD73 is an ecto-enzyme producing extracellular adenosine, promoting breast cancer growth, whereas EZH2 is a methyl-transferase capable of favoring tumor growth by acting as an epigenetic modifier. The dual delivery of such siRNAs had the most significant impact on tumor volume reduction compared with the single ones [13]. Mesoporous silica NPs [49] were used to simultaneously deliver DOX and an siRNA targeting the P-gp, and it was tested against the MCF-7/MDR tumors, a MDR model. Results showed that the drug combination had a synergistic effect in inhibiting tumor growth and overcoming DOX MDR [60]. 

### 3.2. Combined Nanomedicines to Treat Melanoma

For a long time, the standard chemotherapy for melanoma depended on dacarbazine or temozolomide (TMZ). More recently, therapy became more effective owing to the use of rapidly accelerated fibrosarcoma B (BRAF) and mitogen-activated protein kinase (MEK) inhibitors, which are active against BRAF-mutated melanomas. A further step has been immunotherapy with monoclonal antibodies inhibiting immune-checkpoint receptors such as cytotoxic T-lymphocyte antigen 4 (CTLA4) and programmed cell death protein 1 (PD1) [48,61,62]. These can unblock the anti-tumor immune response, which is particularly relevant in this highly immunogenic tumor. Nonetheless, melanoma remains an aggressive tumor with unpredictable responses to chemotherapy and requires improved therapeutic approaches. 

In experimental melanoma, most approaches available to deliver combined nanomedicines make use of liposomes, either passively or actively targeted. 

Arg–Gly–Asp (RGD) and tumor-necrosis-factor-related apoptosis-inducing ligand (TRAIL) is the most relevant ligands employed for active targeting. In the B16-F10 model, liposomes exposing the RGD motif, targeting tumor-overexpressed avβ3 and αvβ5 integrins, were used for the simultaneous delivery of DOX and combretastatin A-4, a potent inhibitor of tubulin polymerization [63], and were highly effective at reducing the tumor growth. In the same melanoma model, intratumoral injection of liposomes loaded with a proapoptotic peptide and an siRNA against the anti-apoptotic molecule Bcl2 [64] increased apoptosis of the tumor cells. TRAIL, which can also be used as a therapeutic agent, and PTX were co-delivered by a liposome system [65,66] because PTX increases the expression of the TRAIL death receptors DR3 and DR4. In this system, TRAIL was grafted to the negatively charged liposome surface, while PTX was encapsulated inside. Moreover, the system TRAIL–PTX was modified by adding the final surface modification with a stearyl chain fused to a pH-sensitive cell-penetrating peptide. This peptide binds the melanoma cells’ integrin receptors αvβ3 and induces the subsequent release of TRAIL in the low-pH tumor microenvironment. This event is followed by the reversion of the surface charge of liposomes and their subsequent internalization. C57BL/6 mice bearing B16-F10 tumors were used to test this therapy, and results showed that the liposomal complex was effective in reducing tumor size and weight, and in increasing mice survival rate, with a lower in vitro IC_50_ than other formulations. 

Among passively targeted liposomes, PEGylated liposomes loaded with DOX (Doxil) have been used in combination with antibodies directed against immune checkpoint inhibitors such as anti-CTLA4 [67]. In a recent work, an anti-CTLA-4 antibody combined with Doxil was highly effective at reducing the growth of B16 melanomas, and induced an increase in the CD8 T/Treg cells ratio in tumor-infiltrating lymphocytes, compared with nonliposomal treatments, without any significant side effects [68,69]. Another approach to unblock the anti-tumor immune response was based on the release of tumor growth factor (TGF)-α-inhibitors combined with high doses of interleukin (IL)-2 [70], loaded in liposomal polymeric gels (nanolipogels), whereas methacrylate-conjugated β-cyclodextrins were inserted inside the liposome, in order to deliver small hydrophobic molecular inhibitors and water-soluble cytokines into the tumor microenvironment. This system was found to promote the activity of natural killer (NK) and CD8^+^ T cells in the tumor microenvironment and to reduce the growth of B16 tumors. 

Advantageously, nanoemulsions for total parenteral nutrition (Intralipid^®,^ BBraun, Melsungen, Germany) were also used to deliver polychemotherapy for melanoma. TMZ; rapamycin (RAP), a selective mammalian target of rapamycin (mTOR) inhibitor; and bevacizumab (BVZ), a humanized anti-VEGF monoclonal antibody, were co-loaded in Intralipid^®^ and tested in vivo on the B16-F10 melanoma mouse model. The drug combination was efficiently loaded in the liquid lipid matrix of Intralipid^®^, including the BVZ monoclonal antibody, leading to decreased tumor growth in vivo with inhibition of tumor angiogenesis and mitotic index [6]. In a similar system, Intralipid^®^ was loaded with TMZ, the kinase inhibitor sorafenib, and a soluble recombinant form of inducible T cell co-stimulator (ICOS-Fc), capable of inhibiting tumor cell migration and angiogenesis and of supporting the anti-tumor immune response. This therapy was effective at inhibiting the growth of mouse melanoma in vivo by exerting a potent anti-angiogenic effect and a complex immunoregulatory activity [7].

Finally, DOX was used in combination with metformin (MET): recently, Song M et al. [71] developed a DOX-MET combined delivery system using pH-sensitive, tumor-targeted NPs including sodium alginate, cholesterol, and FA. This combined system proved to be highly effective in the A375 xenograft melanoma model due to its ability to accumulate in tumor tissue, causing a pan-cell death (apoptosis, necrosis, and pyroptosis) and tumor growth arrest. 

### 3.3. Combined Nanomedicines to Treat Glioblastoma

Glioblastoma multiforme (GBM) is a brain tumor with a very poor prognosis and, despite advancements in surgical therapies, patient survival is below 6% [72]. Currently available chemotherapy is only a palliative care. Indeed, its challenge, besides resistance to chemotherapy, is the need of drugs able to cross the blood–brain barrier (BBB) and to reach the target tissue in the central nervous system. To this aim, active targeting of nanomedicines, either to the BBB, or to the tumor, plays a key role.

Because TMZ is the current standard chemotherapy for GBM, used as an adjuvant after tumor mass resection, several works are focused on improving the chemotherapeutic efficacy of this alkylating compound. Indeed, TMZ can cross the BBB, but its cytotoxicity is highly limited by chemoresistance. Therefore, combined nanomedicines are mainly addressed to overcome TMZ chemoresistance through various mechanisms. Activatory peptides for p53 and TMZ were co-loaded in PLGA NP and functionalized with an RGD peptide [73]. This system has been shown to be effective in reducing tumor growth, induced by intracranial injection of U-87 cells in nude mice, compared with free TMZ. Moreover, PLGA microspheres co-loaded with aspirin and TMZ have also been developed [74]. The microspheres were tested in a subcutaneous LN299 GBM model. Tumor growth was reduced as well as β-catenin, transcription factor 4 (TCF4), protein kinase B (AKT), signal transducers and activators of transcription 3 (STAT3), and proliferating-cell nuclear antigen (PCNA) expression. Polymeric micelles composed of the triblock copolymer (poly(ε-caprolactone), poly(ethylenimine), and PEG, and functionalized with FA, were co-loaded with TMZ and siRNA against B-cell lymphoma 2 (Bcl2). They increased the rat C6 glioma cell apoptotic in vitro response and decreased the glioma growth in rat models, prolonging their survival [20]. In the same tumor model, the PTX + TMZ combination loaded in MPEG-PLGA NPs was found to be more effective at inhibiting tumor growth than the free drugs or the single drugs encapsulated alone [75].

Moreover, DOX-based combined nanomedicines were also employed for GBM, provided that active and/or passive targeting mechanisms should be exploited to improve the drug distribution to the brain. A liposome-based DOX-TRAIL system was used to treat GMB induced with U87 cells to overcome the TRAIL short half-life, DOX cardiotoxicity, and MDR [76]. Liposome-DOX+liposome-TRAIL showed increased anticancer activity compared with the liposomes loaded with the single drugs alone. In order to better deliver DOX to the tumor site, the liposomes were decorated with IL-13 because the IL-13 receptor (L-13 receptor alpha2 protein) is overexpressed in GBM. Results showed that liposomes functionalized with IL-13 displayed an increased ability to release DOX in the tumor site and exerted an improved therapeutic effect compared with unfunctionalized liposomes [77]. 

## 4. Three-Dimensional Human Cell Models 

Cell culture mammalian models have represented an important pillar for the drug discovery process because they provide a fast, simple, and cost-effective tool to avoid large-scale, expensive animal testing. However, classical two-dimensional (2D) cell cultures scarcely mimic the in vivo conditions due to the absence of polarity and microenvironment, among other factors. Three-dimensional (3D) cell cultures have received remarkable attention because they better mimic in vivo features than 2D cultures [78]. Thus, they may be considered a tool to fill the gap between animal studies and clinical trials [79]. Many 3D cell culture models have been set up. Among them, the multicellular tumor spheroids are one of the most used to study cancers in 3D [80]. Here we present a selection of studies on 3D human breast, melanoma, and glioblastoma cell models used to test the anti-tumoral effects of combined nanomedicines. 

Spheroid 3D models of MDA-MB-453 and MDA-MB-231 breast cancer cells were used to test the efficacy of immunoliposomes containing the trastuzumab monoclonal antibodies, targeting HER, plus docetaxel, a taxane-based chemotherapeutic [81]. Immunoliposomes performed better than free trastuzumab plus free docetaxel. These results were also confirmed in vivo in mice, where immunoliposomes showed higher anticancer effects and prolonged survival when compared with the administration of the two free drugs [81]. Ibiyeye et al. [82] generated 3D mammospheres enriched with breast cancer stem cells (BCSCs) responsible for breast cancer chemoresistance [83]. They found that cockle-shell-derived aragonite calcium carbonate nanoparticles (ACNP) loaded with DOX and thymoquinone, a phytochemical with anticancer properties, were more effective at reducing cellular migration, invasion, and inhibition of 3D sphere formation than the free drugs and the single-drug-loaded NPs [82]. Palvai et al. [84] succeeded in co-delivering the hydrophobic drug PTX and the hydrophilic drug CIS in a single poly(isobutylene-*alt*-maleic anhydride) (PMAn) NP. When MCF7 breast cancer spheroids were treated with this nanoformulation, they found an improved breast cancer cell death compared with the free drug combination [84].

A multifunctional NP was set up to deliver two drugs simultaneously, as well as to better penetrate solid tumors, taking melanoma as a model. Albumin NPs loaded with two complementary drugs, riluzole, an inhibitory regulator of glutamate receptors, and curcumin, a phytochemical anticancer agent, were also decorated with collagenase [85]. The collagenase coating together with the combined drug therapy significantly enhanced cytotoxicity in an interesting 3D multicellular spheroid model consisting of C8161+ melanoma cells co-cultured with human foreskin-derived fibroblasts [85].

Concerning glioblastoma, DOX and the multidrug resistance modulator curcumin were simultaneously incorporated in 1,2-distearoyl-*sn*-glycerol-3–phosphoethanolamine-N-[methoxy(polyethylene glycol) 2000] (PEG-PE)-based polymeric micelles to treat 3D spheroids of U87MG glioblastoma cells [86]. Moreover, the polymeric micelles were decorated with an scFv against the glucose transporter GLUT-1, which is significantly overexpressed in many cancer cells and is also distributed in the BBB plasma membranes. Results showed increased efficacy of the drug co-delivery in the functionalized nanoformulations on the U87MG spheroids compared with single-agent-loaded or nontargeted formulations [86]. A different advanced 3D model was set up by Lakkadwala et al. to test the anticancer properties of liposomes containing DOX and ERLO [87]. U98 glioblastoma cells were grown inside a poly (D, l-lactide-co-glycolide)-chitosan (PLGA-chitosan) scaffold, covered with a co-culture of brain endothelial (bEnd.3) and glial cells, mimicking the BBB. This model mimics in vivo conditions, because liposomes must cross the BBB-like barrier before reaching the glioblastoma tumor. Results showed an efficient co-delivery of DOX and ERLO across the BBB-like barrier to the glioblastoma cells, with enhanced cytotoxicity compared with the free-drug treatments [87].

Collectively, these results demonstrate the increased efficacy of drug co-delivery in decorated nanoformulations compared with single-agent-loaded or nontargeted formulations, reinforcing the rationale of using tumor-targeted nanomedicines as a platform for cancer polychemotherapy delivery.

## 5. In Vivo Human Studies 

Research of new therapeutics for cancer treatments is time-consuming and expensive. A new medication takes an average of 7.3 years with a cost ranging from USD 648 million to USD 2.7 billion. Nearly 65% of drugs fail to reach Phase III due to several factors, including the dissimilarities among Phase II and III trials. Indeed, the chances of successful transformation to Phase III depend upon the data quality obtained during Phase I and II clinical trials [88,89]. The overall likelihood of approval is below 10% for new candidates undergoing clinical trials [90], whereas in the case of nanomedicines most failures are due to the above-mentioned scale-up, safety, and efficacy concerns [91]. Noteworthy is that the most relevant part of clinical trials with nanomedicines in terminal cancers regards off-label use of already marketed liposomal formulations, either alone or in combination [39,48]. The NAPOLI-1 clinical trial tested nanoliposomes with irinotecan, 5-FU, and folic acid (leucovorin) in monotherapy or in combination in patients with pancreatic ductal adenocarcinoma [92,93]. Patients treated with the combination therapy showed a higher survival rate and fewer side effects than those treated with monotherapy, and this combination therapy has been approved by the Food and Drug Administration (FDA) for the treatment of patients with metastatic pancreatic ductal adenocarcinoma. 

Here below, the main issues that new combined nanomedicines can encounter while entering clinical trials are described in Table 1 [83,92,93,94,95,96].

### 5.1. Animal-to-Man Gap

Numerous studies have tried to clarify this relevant aspect. The most interesting finding is that there are differences in function between rodents and humans [97]. Indeed, results from four different mouse models and three types of human cancer cells were analyzed. The data obtained with imaging techniques were used for simulation and mathematical modeling. This pointed out that transendothelial pathways are the dominant mechanism of NPs tumor extravasation, which is not directly related to the frequency of lacunae in tumors. Gaps, as demonstrated with transmission electronic microscopy and 3D acquisitions, were not so numerous, thus making the possibility of passive transport rare [98]. To better clarify these observations, studies were performed on cancer patients in order to identify possible predictive markers of the EPR effect [99,100]. The results indicated that the EPR effect is variable, depending on species and tumors. Further research is therefore necessary to clarify the transport mechanism of the nanocarriers. However, several studies were restricted to a single experimental model, making it difficult to mimic the real effects in humans, even if most of these studies seem to indicate a greater relevance of the EPR effect in animals compared with humans [101]. Among these studies, carried out with various technological platforms of nanocarriers, some are in phase 1 and are directed, for example, at colon cancer, breast cancer, and B-cell non-Hodgkin lymphoma; others are in phase 2, such as those for basal cell carcinoma and prostate and HER2-positive metastatic breast cancer; some have reached phase 3, such as those concerning small-cell lung cancer (SCLC). Some nanomedicines have already been approved by the FDA for breast cancer, pancreatic cancer, and NSCLC, the most important being PTX delivered in albumin NPs [102]. Liposomes have also been approved, such as those for the delivery of vincristine sulfate against acute lymphoblastic leukemia [103]; of cytarabine in lymphomatous malignancies [104]; of DOX in ovarian cancer, multiple myeloma [105], and HIV-related Kaposi’s sarcoma [105,106]; and also of polymer-protein conjugate-carrying L-asparaginase for leukemia [107]. 

Instead, the efficacy on metastases is rather dismissed, even if it is crucial due to their high frequency in malignant tumors. Therefore, although an effective single approach to these concerns is hard to find, new techniques, such as organ/tumor-on-chip tools, may be considered as potential solutions to broaden the knowledge of the use of nanocarriers in cancer patients [108,109,110]. 

### 5.2. Interaction with the Endogenous Environment

Once systemically injected, the nanocarriers contact the serum and the cellular proteins, which wrap them and form a new structure, whose components are defined as the protein corona [111,112,113]. The high binding affinity of the proteins for the NPs causes the formation of an inner layer named hard corona, while soft corona is an outer layer formed by loosely bound proteins. This causes displacement among the proteins, which will eventually lead to the total replacement with those having a higher affinity [112]. Therefore, the protein corona interposed between the NPs and the biological environment regulates their reciprocal interactions and, to a certain extent, also their medical use, having an impact on the biodistribution, toxicity, and targeting efficacy of the NPs. 

Proteomic methods, which analyze proteins in serum and tumor cells, can be used to study the nanoparticle/protein interactions in order to improve the understanding of this phenomenon and to find biomarkers useful for prognosis [113]. The most commonly used techniques include surface plasmon resonance, isotherm microcalorimetry, mass spectroscopy quantitative analysis, liquid chromatography coupled to mass spectroscopy, and sodium dodecyl sulphate–polyacrylamide gel electrophoresis [114]. These techniques may also facilitate the discovery of new molecular targets and the identification of specific molecules useful for improving the performance of anticancer nanomedicines. 

Within the files on nanomedicines, one of the main limits is the fragmentation of published studies, which are often contradictory or incomplete and therefore biased. Frequently, in vitro systems do not truly mimic the natural in vivo environment, because the selected cell models replicate only one experimental concern among all those that should have been considered. This further expands the valid and genuine toxicity concerns of nanoscale entities in human patients and healthy volunteers. The NPs’ surface-driven biodistribution is influenced by various phenomena, such as nonspecific absorption or removal by the reticuloendothelial system or perturbations in the biological barriers [115]. 

Many of these aspects undoubtedly also depend on the administration route and related elimination pathways from the body. The transdermal route, for example, sees elimination mainly through skin exfoliation or sweating. Intravenous administration, instead, involves blood delivery and metabolism by several organs, such as the heart, kidneys, spleen, bone marrow, liver, and lymphatic system, as well as platelets, monocytes, and endothelial cells, with a prevalent elimination in the urine and feces. The same fate occurs for inhalable formulations, either nasal, tracheobronchial, or alveolar. Oral formulations, interacting with the gastric tract, are also involved with the blood and lymphatic pathways [116].

### 5.3. Unexpected Toxicity

The issue of nanocarriers’ carcinogenicity in humans has been scarcely explored, even if the results of qualitative studies point the attention to possible features, including small size, absorption capacity, retention time and duration, biodistribution, and interactions at the subcellular or molecular level [117]. 

Concerning the structures, polymeric or metallic NPs coated with organic, natural, or nature-mimicking polymers have been observed to be nontoxic and highly biocompatible for pharmaceutical applications [118,119,120,121,122]. It has recently been ascertained that size and shape have the greatest influence on the toxicological characteristics of NPs. The higher toxicity is associated with large sizes, regardless of shape (nanospheres, hollow polymeric nanovesicles, spherical polymeric micelles, nanorods, nanostars, or nanoworms) [123]. The effect of surface coatings has also been explored, with the use of polymers, molecular coatings, and lipid bilayers to protect the therapeutic molecules; modulate their release; and ensure their stability, hemocompatibility, and biocompatibility in the physiological media [124,125,126]. NPs coated with gelatin or casein polymers [127], chitosan [128], eudragit polymers [129], polyacrylic acid [125], acrylic polymers [130], silsesquioxanes and PEG [131], lipid coatings, etc. [125] have been widely employed to increase biocompatibility with cell lines and tissues. 

Numerous parameters were studied to determine the safety and toxicity of nanocarriers: cytotoxicity, using cell proliferation, morphology, and vitality studies [132]; genotoxicity, aimed at determining DNA breaks, chromosomal damage, or altered bases [133,134,135]; levels of reactive oxygen species as the depletion of antioxidant capacities [136,137,138]; cytokine production [139,140]; carcinogenicity in tumor-prone transgenic models [141,142]; and hepatotoxicity, using bioprinting or organoid techniques that minimize the use of animals and truly approach preclinical conditions [143]. Moreover, omics techniques such as metabolomics, proteomics, and genomics have been used [144].

Finally, because both the nanocarriers and the drug cargoes are extrinsic molecules, they can induce the activation of reticuloendothelial cells in the liver, spleen, or lungs, which would shorten their half-life [145]. In addition, small nanocarriers may pose risks because of their possible accumulation across barriers. Recent evidence suggests that cell membranes, organelles, and DNA are sensitive to free radicals generated by NPs [146,147]. Furthermore, nanomaterials could stimulate an immune response by reacting with cell surface receptors [148,149]. 

Therefore, when designing a nanocarrier, improvement of efficacy, prevention of resistance, and reduction of side effects should be considered together. Despite much research into anticancer nanomaterials, inherent problems are not resolved yet. 

## 6. Unconventional Nanocarriers

In addition to traditional nanocarriers designed to work as vehicles for drug combinations, promising unconventional nanocarriers may be exploited. These include physiological vesicles such as extracellular vesicles (EVs), which are endowed with intrinsic therapeutic and/or targeting properties exploitable for drug/nucleotide delivery. Moreover, among protein NPs, polyhedra represent a relevant innovation, given their ability to cage a large amount of protein drugs while retaining their functionality.

### 6.1. Extracellular Vesicles

**Figure 3:**
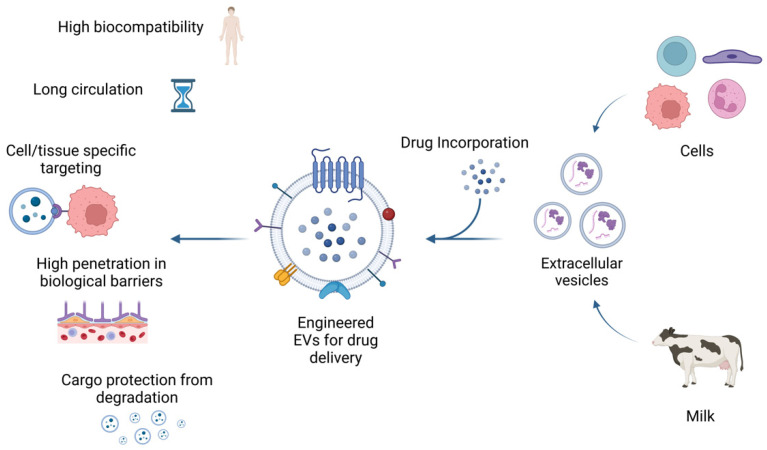
Biological properties of extracellular vesicles. EVs released from cells or isolated from bovine milk can be used for drug delivery carriers. Moreover, EVs loaded with therapeutic agents can be engineered to enhance their targeting of specific cells or tissues. EVs have high biocompatibility, low immunogenicity, and the ability to cross biological barriers such as the BBB. In addition, EVs can extend the drug half-life by protecting their cargo from degradation. This figure was created with BioRender.

EVs are vesicular structures embedded in a lipid membrane, which are released from most cells and can carry different kinds of physiologic cargoes such as lipids, nucleic acids, and proteins [150]. When EVs are released, these cargoes are delivered to local and distant cells, playing an important role in intercellular communication [151]. EVs can be differentiated by both size and biogenesis and include exosomes, microvesicles, and apoptotic bodies [152,153]. Apoptotic bodies are formed as a consequence of programmed cell death and are the largest EVs, sized 500–4000 nm [154]. Microvesicles (MVs) have sizes ranging 100–1000 nm and are generated from outward membrane budding [155]. MVs and apoptotic bodies are differentiated by their size, formation, content, and surface antigen expression. Exosomes are generated by inward budding of endosomes and are the smallest EVs, sized 40–120 nm [156] (Figure 3).

EVs play an important role in the homeostasis of healthy organisms and in regulation of the inflammatory/immune response due to their cargo of several chemokines, cytokines, and other immune molecules [157,158]; they are also implicated in several diseases such as neurodegenerative diseases, autoimmune diseases, and heart disease [159,160,161,162]. Moreover, tumor-cell-derived EVs are involved in several aspects of cancer progression, such as angiogenesis, EMT, cell proliferation, and drug resistance [163]. 

EVs have been proposed to be an ideal drug delivery system due to their high biocompatibility, prolonged life span, low immunogenicity, and low toxicity [164,165,166,167]. The protein profile on the EV surface allows them to target specific tissues and promotes their binding to specific target cells. When EVs bind to the target cells, they can activate several signaling pathways to generate specific responses, or they can release their cargoes into the target cells upon endocytosis or direct membrane fusion [168]. 

Tumor-derived EVs can be useful for cancer therapy due to their ability to accumulate in the tumor tissue, and they can be engineered to enhance their targeting of tumor cells [168,169,170,171]. EVs can be loaded with drugs using several direct methods, such as incubation, sonication, and electroporation. As an alternative, loading with indirect methods uses the drug to treat parent cells, which then release EVs already loaded with the drug [172]. CD47-mediated protection of exosomes from phagocytosis prolongs their blood circulation [173,174]. Due to their liposolubility, EVs can also cross physical barriers such as the BBB, helping to solve a key problem related to the treatment of brain cancer [175].

Studies in mice bearing ovarian or breast cancer showed that DOX uploaded in EVs displays decreased cardiotoxicity and an improved therapeutic effect [176,177]. EVs carrying CIS significantly enhance the cytotoxic effect against drug-resistant A2780/DDP cells [178]. Treatment with GEM loaded into EVs derived from Panc-1 cells achieves the suppression of tumor growth and no recurrence in mice bearing pancreatic ductal adenocarcinoma [179]. In colon and gastric cancer, EVs carrying anti-miRNA and chemotherapeutics have been shown to reverse drug resistance and to improve chemotherapy performance [180,181,182]. EVs derived from brain endothelial cells and loaded with PTX and DOX were able to pass across the BBB and cause cytotoxic effects on brain tumor cells, in addition to being associated with decreased cardiac toxicity [176,183]. 

Another interesting possibility is the use of exosomes isolated from bovine milk as a drug delivery system, showing advantages in terms of cost, biocompatibility, and physical and biological stability [184]. Oral delivery of PTX loaded in milk exosomes showed significant tumor growth inhibition in human lung tumor xenografts and a continuous drug’s release for up to 2 days [185]. Similar anti-tumor efficacy was detected in ovarian cancer xenografts by oral treatment with milk exosomes loaded with PTX and anthocyanidins [186]. 

### 6.2. Protein NPs Delivery System

Protein-based NPs are a promising delivery system for anticancer drugs [187]. In particular, owing to their minimal toxicity and immunogenicity, serum proteins have gained considerable attention in the formulation of several delivery systems for anticancer drugs [188]. Serum proteins such as albumin, gelatin, and transferrin are widely used as the carriers for small-molecular therapeutic drugs and imaging agents [189,190]. Furthermore, proteins are good candidates for conjugation with drugs, as they provide good pharmacokinetics as well as increased accumulation in the cancer tissue [166]. Due to the amine and carboxyl groups’ reactive residues, serum proteins are more suitable for chemical modifications to conjugate therapeutic drugs, imaging probes, as well as ligands for active targeting [191]. 

Albumin NP loaded with PTX showed a promising efficacy in mouse cancer treatment in vivo owing to the increased expression of albumin-binding proteins in tumors such as colon cancer and glioma [192], interest in which is increased by the ability of these NPs to cross the BBB [193]. Ferritin, too, emerged as an excellent and promising protein-based nanocage owing to its unique architecture, surface properties and high biocompatibility. Ferritin nanocages may ensure a proper drug delivery and release by exploiting natural recognition of the Transferrin Receptor 1, which is over-expressed on tumor cells [194,195,196,197]. 

**Figure 4:**
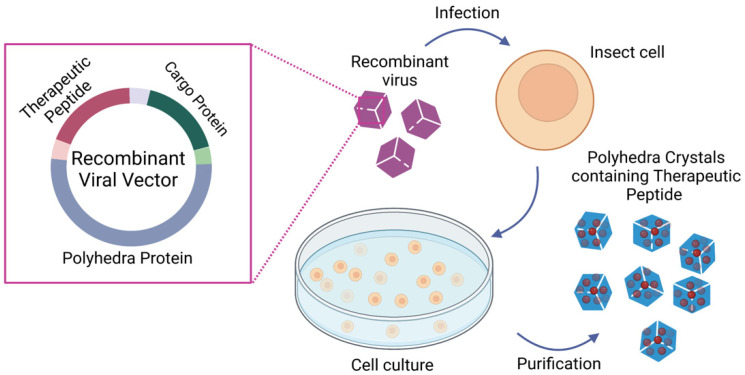
Polyhedrin delivery system (PODS) production: Schematic representation of the immobilization of therapeutic peptides into polyhedra crystals. Briefly, a recombinant viral vector carrying polyhedra protein, cargo protein, and the therapeutic peptide is used to transduce insect cells. The infected cells produce polyhedra crystals containing the therapeutic peptide, which are purified with differential centrifugation. The mechanical properties of polyhedra allow the purification of polyhedra crystals without compromising the therapeutic peptide functionality. This figure was created with BioRender.

An intriguing, novel approach to produce protein NPs takes advantage of the polyhedra (also called polyhedrin proteins) produced by baculoviruses and cypoviruses. Polyhedra have a protective function, forming a protective crystal around the virus, and can resist solubilization, conferring to the viruses the ability to remain viable for many years outside the host [198]. These properties have attracted the interest of the medical scientific community in using these proteins as delivery systems for protein drugs because co-expressed recombinant proteins can be incorporated in polyhedra by taking advantage of the self-assembling property of these proteins in infected cells [199,200,201], allowing a large amount of protein to be caged within polyhedrin crystals while retaining their functionality [201,202,203,204]. Therefore, polyhedrin proteins can be used as a delivery system (polyhedrin delivery system, PODS) for many therapeutic peptides and also can be applied to cancer therapy (Figure 4). An example is the successful inclusion of endostatin into polyhedrin microcrystals, whose local administration inhibited both angiogenesis and tumor growth in SCC IIV squamous cell carcinoma in mice [205]. Interestingly, Wendler et al. explored the potential of PODS for delivering functional cytokines to monocytes and macrophages. They showed that PODS particles are very efficiently phagocytosed and that cargo cytokines avoid or resist the harsh conditions of phagolysosomes and enable secretion by macrophages. These results suggest the potential utility of PODS in Trojan horse drug delivery strategies for the treatment of cancer [206].

## 7. Conclusions

Combined nanomedicines show several advantages in cancer therapy. Most relevant applications concern chemoresistant tumors, such as triple-negative breast cancer; highly metastasized tumors, such as melanoma; and tumors in which drug targeting is hampered by biological barriers, such as the BBB in the case of glioblastomas. Indeed, multiple drugs, acting on distinct mechanisms, can be co-loaded in combined nanomedicines, with both physicochemical and pharmacological purposes. Physicochemical purposes include overcoming solubility issues and separating incompatible compounds into physically distinct environments. Synergy of action, instead, is the general pharmacological goal, including the mechanisms for overcoming MDR. This can be achieved by means of the controlled drug release and the targeting capability of the nanocarriers. Within this context, hybrid nanomaterials represent a new paradigm, owing to their composite structure, drug-loading capability, and bioengineering-based approach, despite that, so far, studies regarding their biosafety in animal models are still premature. Emerging/unconventional EVs and polyhedra show promising advantages, due either to intrinsic therapeutic and/or targeting properties or to the ability to carry a large amount of bioactive protein drugs. However, several limitations still affect the use of combined nanomedicines in cancer therapy. In addition to scale-up and toxicological concerns, strictly related to the production method employed, dose timing among the delivered drugs is crucial in order to determine either synergy or antagonism. Indeed, the co-encapsulation process follows its own ratios that often do not match the ones required for synergism. Finally, reproducibility issues are caused by the confinement of multiple drugs within the nanocarrier core, which might either cause biochemical alterations of the loaded compounds or decrease the nanocarrier’s colloidal stability. Therefore, to date, despite the huge number of studies in the literature about combined nanomedicines, only Vyxeos liposomes have gained approval for clinical usage.

## Figures and Tables

**Table 1:** Clinical trials using nanomedicine for combination therapy.

Material Type	Description	Approved Indication	Status	Reference
Liposome	Cytarbine, Daunorubicin	Acute Myeloid Leukemia	Approved by FDA and EMA	[94]
Polymer Conjugate	Cetuximab loaded with Stomatostatin analogue	Colon Cancer	Phase I	[83]
Protein	Paclitaxel/Rituximab Co-coated drug	B cell non-Hodgkin Lymphoma	Phase I	[83]
Pegylated Liposome	Doxorubicin Transtuzumab	Her 2 positive Metastatic Breast Cancer	Phase II	[83]
Protein	Paclitaxel, Carboplatin, Temozolomide, Bevacizumab	Malignant Melanoma	Phase II	[95]
Nanoemulsion	Photosensitizer, HAL, BF-200 ALA, MAL In PDT Theraphy	Basal Cell Carcinoma	Phase II	[83]
Protein	Paclitaxel, Carboplatin, Pembrolizumab	NSCLC	Phase III	[96]
Liposome	Irinotecan, 5-fluorouracil, Leucovorin	Pancreatic Adeno Ductal Carcinoma	Phase III	[92,93]

## Data Availability

Not applicable.
